# Automated molecular structure segmentation from documents using ChemSAM

**DOI:** 10.1186/s13321-024-00823-2

**Published:** 2024-03-12

**Authors:** Bowen Tang, Zhangming Niu, Xiaofeng Wang, Junjie Huang, Chao Ma, Jing Peng, Yinghui Jiang, Ruiquan Ge, Hongyu Hu, Luhao Lin, Guang Yang

**Affiliations:** 1 College of Life Sciences, Zhejiang University, Hangzhou, 310058 China; 2 MindRank AI Ltd., Hangzhou, 310000 China; 3 Hangzhou Institute of Advanced Technology, Hangzhou, 310000 China; 4 Hunan University of Medicine, Huaihua, 4180001 Hunan China; 5 Hangzhou Dianzi University, Hangzhou, 310000 China; 6 Xingzhi College, Zhejiang Normal University, Lanxi, China; 7 Department of Pharmacy, The 910th Hospital of the Joint Logistics Support Force of the Chinese PLA, Quanzhou, 362000 Fujian China; 8 Bioengineering Department and Imperial-X, Imperial College London, London, W12 7SL UK; 9 National Heart and Lung Institute, Imperial College London, London, SW7 2AZ UK; 10 School of Biomedical Engineering & Imaging Sciences, King’s College London, London, WC2R 2LS UK

## Abstract

Chemical structure segmentation constitutes a pivotal task in cheminformatics, involving the extraction and abstraction of structural information of chemical compounds from text-based sources, including patents and scientific articles. This study introduces a deep learning approach to chemical structure segmentation, employing a Vision Transformer (ViT) to discern the structural patterns of chemical compounds from their graphical representations. The Chemistry-Segment Anything Model (ChemSAM) achieves state-of-the-art results on publicly available benchmark datasets and real-world tasks, underscoring its effectiveness in accurately segmenting chemical structures from text-based sources. Moreover, this deep learning-based approach obviates the need for handcrafted features and demonstrates robustness against variations in image quality and style. During the detection phase, a ViT-based encoder-decoder model is used to identify and locate chemical structure depictions on the input page. This model generates masks to ascertain whether each pixel belongs to a chemical structure, thereby offering a pixel-level classification and indicating the presence or absence of chemical structures at each position. Subsequently, the generated masks are clustered based on their connectivity, and each mask cluster is updated to encapsulate a single structure in the post-processing workflow. This two-step process facilitates the effective automatic extraction of chemical structure depictions from documents. By utilizing the deep learning approach described herein, it is demonstrated that effective performance on low-resolution and densely arranged molecular structural layouts in journal articles and patents is achievable.

**Supplementary Information:**

The online version contains supplementary material available at 10.1186/s13321-024-00823-2.

## Introduction

Extracting chemical structure depictions constitutes a fundamental task in chemistry and related disciplines. This process involves identifying and capturing graphical representations of chemical structures, including molecular formulas, chemical reactions, and molecular diagrams. Traditionally, researchers and practitioners have relied on manual extraction, a process that is labor-intensive, time-consuming, and susceptible to human error.

The segmentation process entails identifying graphical representations of chemical compounds within documents and abstracting these into formats conducive to easy processing and analysis. This task poses challenges due to the diversity and complexity of chemical structures, coupled with the variability in their graphical representations across various documents. To tackle these challenges, a range of approaches has been proposed, encompassing both rule-based methods [1–9] and machine learning-based methods [10–12]. Rule-based methods depend on predefined rules and heuristics to identify chemical structures within documents. For instance, the open-source OCR tool OSRA incorporates a rule-based page segmentation algorithm. This mechanism identifies chemical structure depictions by analyzing the dimensions of a rectangular bounding box around regions of interest and the ratio of the black pixels to the total area of the rectangle [8]. This class of chemical structure depictions was also called ‘feature density’ in the open-source tool ChemSchematicResolver (CSR) [13] and DECIMER-Segmentation [18].

Despite the hand-coding of many specific rules, rule-based methods still struggle in various situations, including wavy bonds, overlapping lines (e.g., bridges), limited flexibility, difficulty in handling variability, sensitivity to noise and errors, maintenance overhead, and lack of generalization. In contrast, machine learning-based methods utilize statistical models and algorithms to learn the patterns of chemical structures from training data. For instance, the open-source tool ChemSchematicResolver (CSR) [13] is capable of segmenting images containing only labels and chemical structure depictions. Classification of objects into labels or structure depictions employs k-means clustering based on so-called “feature density”. Feature density was indicated by the ‘skeletonized-pixel ration’ in CSR. This ration value could be used as a crude measure of the ‘density of features’ of the figure. Generally speaking, when this density is high for the entire figure, it is likely to be more crowded with regions of chemical structures. However, CSR [13] cannot handle scanned pages or images containing objects other than structure depictions and labels.

Deep learning-based approaches have recently gained significant attention for their ability to learn complex patterns and representations from data [11, 14–16]. In 2019, Staker et al. developed a chemical structure segmentation procedure using a deep learning method [15]. Unlike the feature density-based approaches used by OSRA [8] and CSR [13], Staker et al. employed a U-Net model to tackle the segmentation challenge [17]. Each image undergoes multiple processing steps at different resolutions, with the model’s generated masks being averaged. The model was trained on a semi-synthetic dataset, where OSRA identified bounding boxes around potential chemical structure depictions across various publications and patents. Subsequently, these areas were excised from the original documents and substituted with structures from publicly available datasets. For data augmentation, the images underwent random modifications during training, such as binarization and brightness adjustments. However, reports of independent segmentation accuracy are scarce, with overall accuracy for the entire segmentation and structural resolution process across various datasets ranging from 41 to 83% [15]. Later, in 2021, Kohulan et al. developed a toolkit named DECIMER-Segmentation [18], based on Mask R-CNN for detecting and segmenting chemical structures from scientific literature [19]. However, DECIMER-Segmentation cannot ensure that each segmentation represents a pure single chemical structure; segments may include non-molecular parts, such as arrows from chemical reactions, additional lines from tables, and other labels. Furthermore, some fonts within the chemical structures may be overlooked, leading to inaccurate or incorrect chemical structures. Therefore, their segmented images require further segmentation or even manual verification to ensure structural accuracy before they can be transformed into right SMILES or chemical graph data. Given the diversity of approaches, we have compiled Table 1. This table aims to provide a clearer understanding of the methodologies employed by various models.

**Table 1:** Comparative summary of chemical structure segmentation models and methodologies

Model	Approach	Methodology or model
Staker et al. [15]	Deep learning-based	U-Net model
DECIMER-segmentation [18]	Deep learning-based	Mask R-CNN
SwinOCSR [12]	Deep learning-based	Transformer
MolMiner-ImgDet [20]	Deep learning-based	MobileNetV2
OSRA [8]	Rule-based	Custom function with feature density
CSR [13]	Rule-based	K-means clustering with feature density
ChemSAM [21]	Deep learning-based	SAM + adapter

**Fig. 1:**
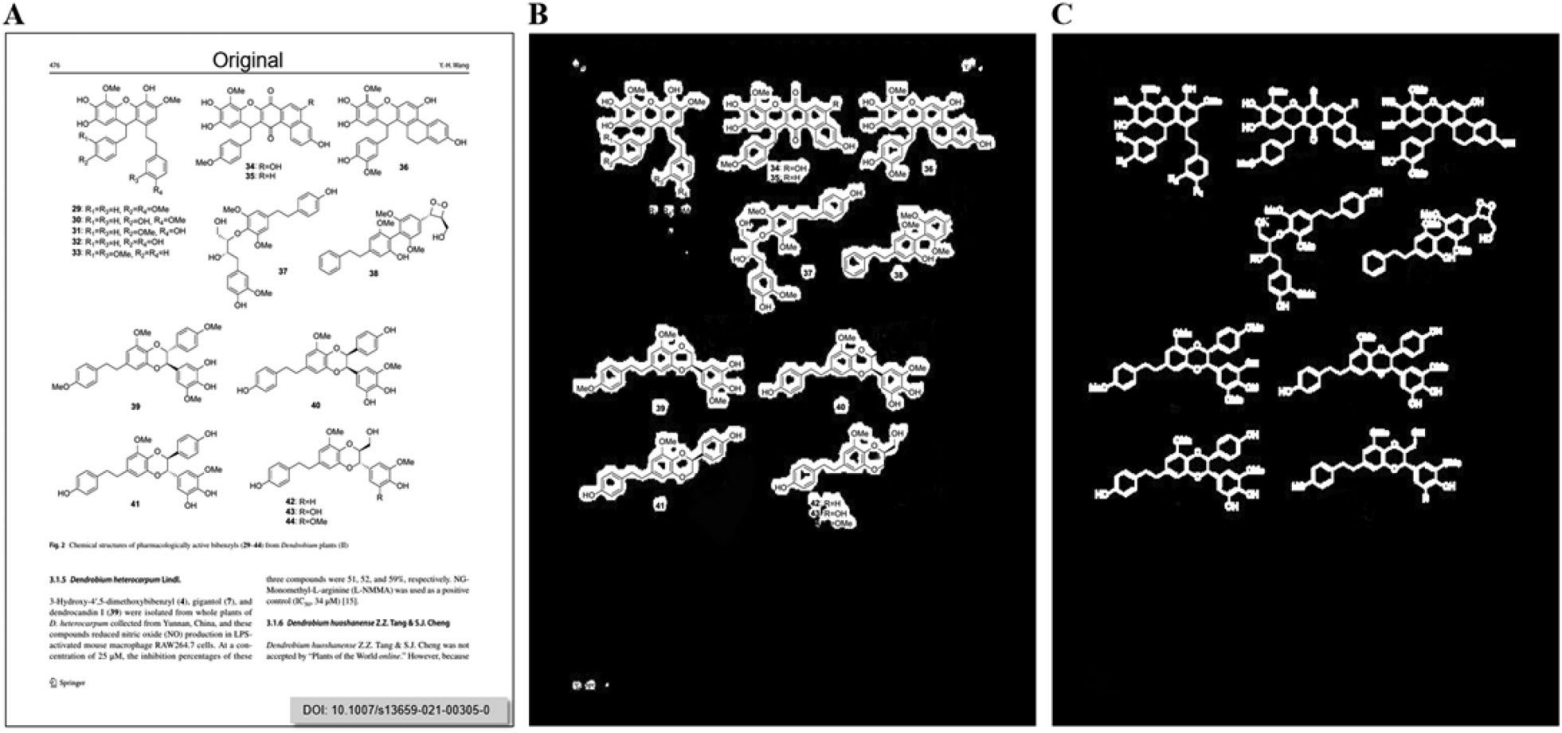
Illustration of ChemSAM’s segmentation process. **A** Displays the original page being analyzed. **B** Shows the output from the initial detection phase, where ChemSAM generates masks to delineate the positions and shapes of chemical structures. **C** Demonstrates the post-processing step, refining potentially incomplete or redundant masks to accurately represent chemical structures, highlighting the model’s capability in enhancing segmentation accuracy

In 2023, the “Segment Anything Model (SAM)” emerged, attracting significant attention as a versatile and powerful vision segmentation model [22]. Despite its robust performance on natural images, the application of the SAM model to chemical structure segmentation has not been previously reported. It might fail in chemical structure segmentation without careful optimization of network architecture and weight refinement. In the ChemSAM project, we extended the SAM model’s capabilities to the chemistry domain, marking a significant step toward the ultimate goal of “Segment Anything.” To our knowledge, we are the pioneers in proposing an adaptation approach for general chemical structure segmentation and automating the extraction process from patent documents and scientific literature. We also incorporated domain-specific chemical knowledge in the adapter’s design. The algorithm comprises two main stages: initially, during the detection phase, ChemSAM generates masks delineating the positions and potential shapes of chemical structures from an input image. The mask may not completely cover the chemical pixels, as displayed in Fig. 1B. This is followed by a mask post-processing procedure, wherein potentially incomplete masks and redundant masks are refined to accurately represent the chemical structure, as shown in Fig. 1C. To ensure widespread availability of ChemSAM [21], the main codes have been published. Our application is also accessible via email request, and we are seeking further collaboration.

## Materials and methods

### Implementation

The ChemSAM Segmentation backend mechanism was developed in Python 3 using Pytorch [23]. It was trained on a Linux system, but its inference can be run on Windows, macOS and Linux systems. It primarily involves the recognition of chemical structure diagrams through a deep learning model and the subsequent post-processing of the resulting masks.

The implementation details of the key elements, along with the complete workflow that accepts PDF documents or image files as input and returns the segmented chemical structure diagrams as output, are described below.

### Deep learning method

**Fig. 2:**
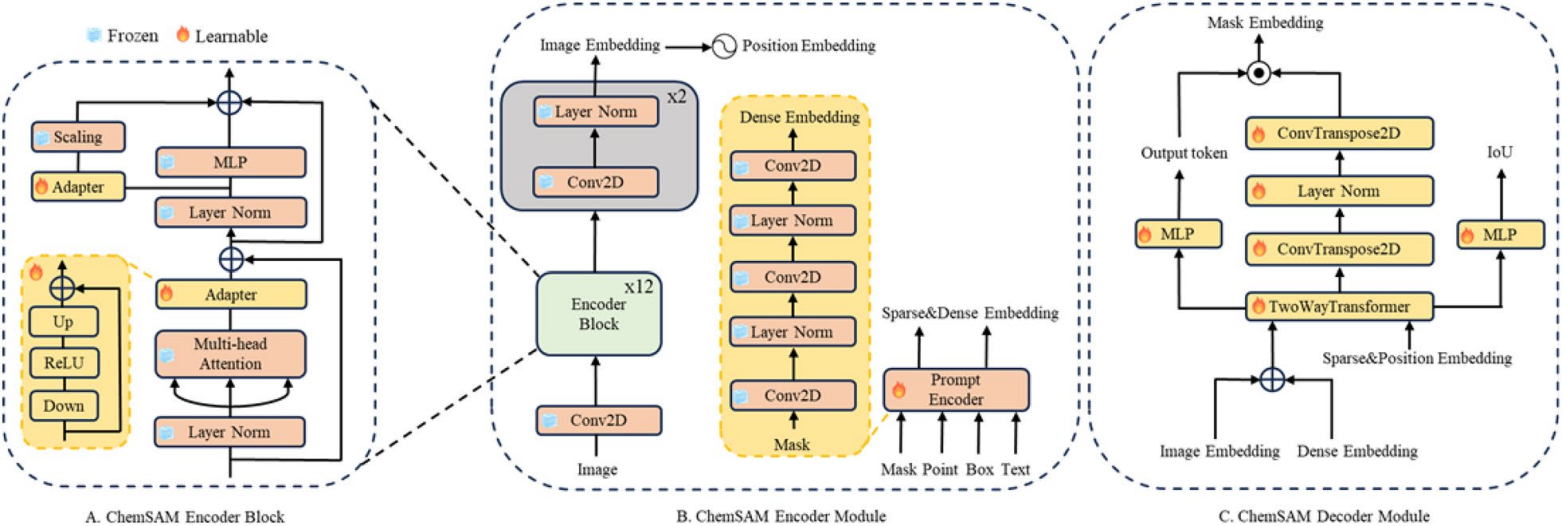
Chemical segment anything model (ChemSAM) overview and adaptions

The ChemSAM network architecture comprises three primary components: an image encoder, a prompt encoder, and a mask decoder. As depicted in Fig. 2B, the image encoding process within ChemSAM involves several steps. Initially, the image undergoes 2D convolution. Subsequently, it is processed through 12 encoder block layers, each featuring two adapters to integrate chemical domain knowledge. Ultimately, the final image embedding is achieved by applying two layers of 2D convolution and regularization. Specifically, the image encoder block utilizes a pre-trained standard Vision Transformer (ViT) [24], trained using a Masked Autoencoder (MAE) [25]. Figure 2A illustrates an encoder block, detailing that the first adapter follows the multi-head attention phase, and the second adapter precedes the MLP layer in the subsequent pathway.

In the ChemSAM model, the prompt encoder is divided into two main components: dense prompts (masks) and sparse and positional prompts (points, boxes, text). Specifically, dense prompts, such as masks, are embedded using 2D convolution and regularization techniques. Sparse prompts, including points and boxes, are encoded using positional encodings [26], subsequently combined with learned embeddings for each prompt type. Furthermore, free-form text is processed using a pre-existing text encoder from CLIP [27].

Furthermore, the mask decoder efficiently maps the image, dense, and sparse and positional embeddings to a mask. As illustrated in Fig. 2C, the process starts with an element-wise summation of the dense embedding and the image embedding. This combination draws inspiration from Transformer segmentation models [28], featuring a modified standard Transformer decoder with a dynamic mask prediction head [29]. Simultaneously, the learned output token embedding is inserted into the set of cue embeddings, to be utilized in the decoder’s output, akin to the [class] token as mentioned in reference [24]. Subsequently, the image embedding is up-sampled by a factor of 4× using two transposed convolutional layers, yielding a representation that is downscaled relative to the original image. Meanwhile, the image embedding is then further up-sampled, and an MLP (Multi-Layer Perceptron) maps the output to a dynamic linear classifier. The intersection over union (IoU) thresholds calculates the mask’s foreground probability at each image location.

In addition, rather than fully fine-tuning all parameters, we chose to keep the pretrained SAM [22] parameters frozen and introduced several adapter modules at specific positions, as illustrated in Fig. 2. The Adapter module, designed as a bottleneck model, consists of a sequence of operations: down-projection, ReLU activation [30], and up-projection. The down-projection step reduces the embedding’s dimensionality via a simple MLP layer, whereas the up-projection step restores the compressed embedding to its original dimension with another MLP layer. Outputs from the decoder network are scaled with a sigmoid activation function, normalizing values between 0 and 1.0. This facilitates probability-based predictions for pixel-wise mask labels, indicating whether pixels correspond to molecular structures.

### Datasets and training

ChemSAM training utilized synthetic data derived from self-collected real-world datasets. We devised a systematic approach to generate training data for the automatic identification of optical compound structural formulas. Initially, we gathered 50 PROTAC-related patents and 41 papers from publicly accessible patent databases (such as Google Patent, EPO) and academic databases (such as Google Scholar). These patents and papers were divided and saved as individual pages. We manually filtered out pages, retaining only those without chemical structure data, resulting in a total of 550 base pages, comprising 277 patent and 273 paper pages.

**Fig. 3:**
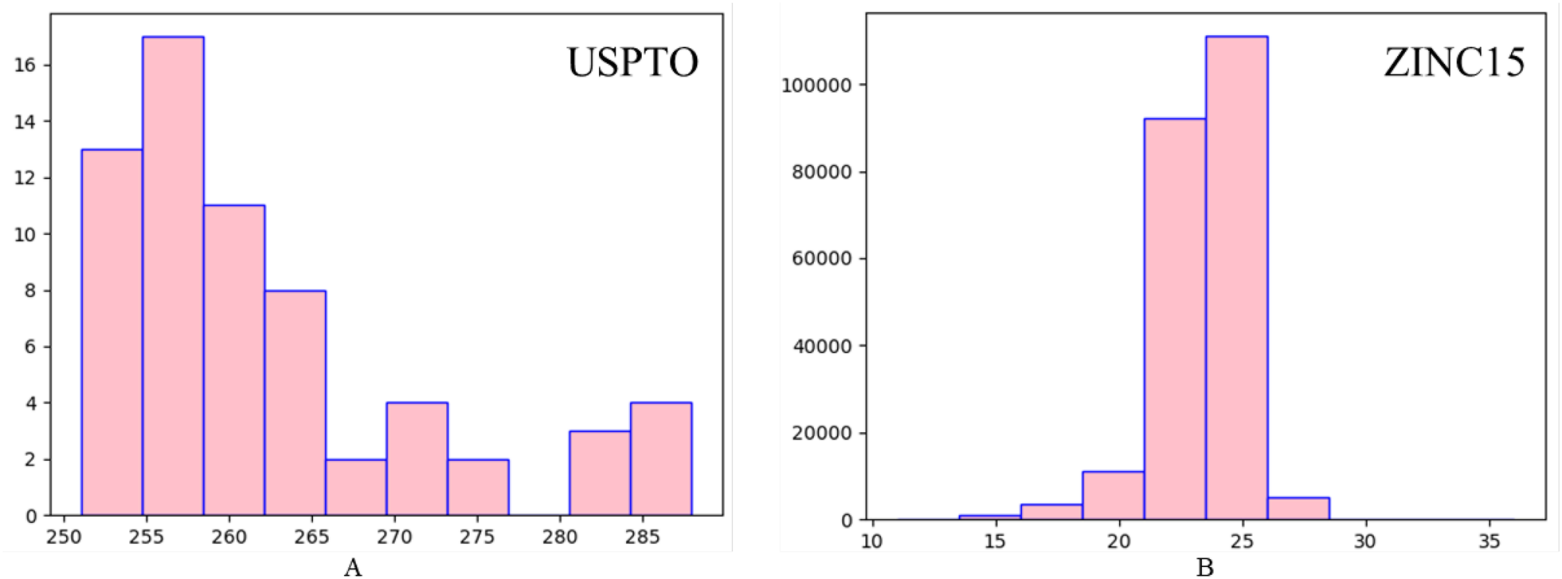
The distribution of used source real-world data. Among them, the abscissa represents the number of atoms in the molecule, and the ordinate represents the number of molecules

For the molecular data, molecules were sourced from the United States Patent and Trademark Office (USPTO) [31] and drug-like molecules from the ZINC15 database [32], with the complete atomic distribution depicted in Additional file 1: Fig. S1. Specifically, to mimic real-world scenarios that involve the clustering of multiple molecules in specific areas of a page, molecular files were carefully selected from the USPTO dataset. As shown in Fig. 3A, the selection criterion was focused on mol files having a total atom count within the dataset ranging from 250 to 300, including non-heavy atoms such as hydrogen. This selection process guaranteed that the selected molecules closely mirror real-world data, featuring coordinates in close proximity. For the ZINC15 dataset, 1 to 6 individual molecules were randomly positioned on each page, with care taken to ensure no overlap between them and adherence to a total number of heavy atoms ranging from 15 to 28 as shown in Fig. 3B. Additionally, a total of 8764 non-molecular structure images were collected from patents and papers as negative samples. Similar to the molecules, the negative sample images were randomly placed on the pages, with measures taken to ensure no overlap with the molecules, to mimic the layout of real-world page.

**Fig. 4:**
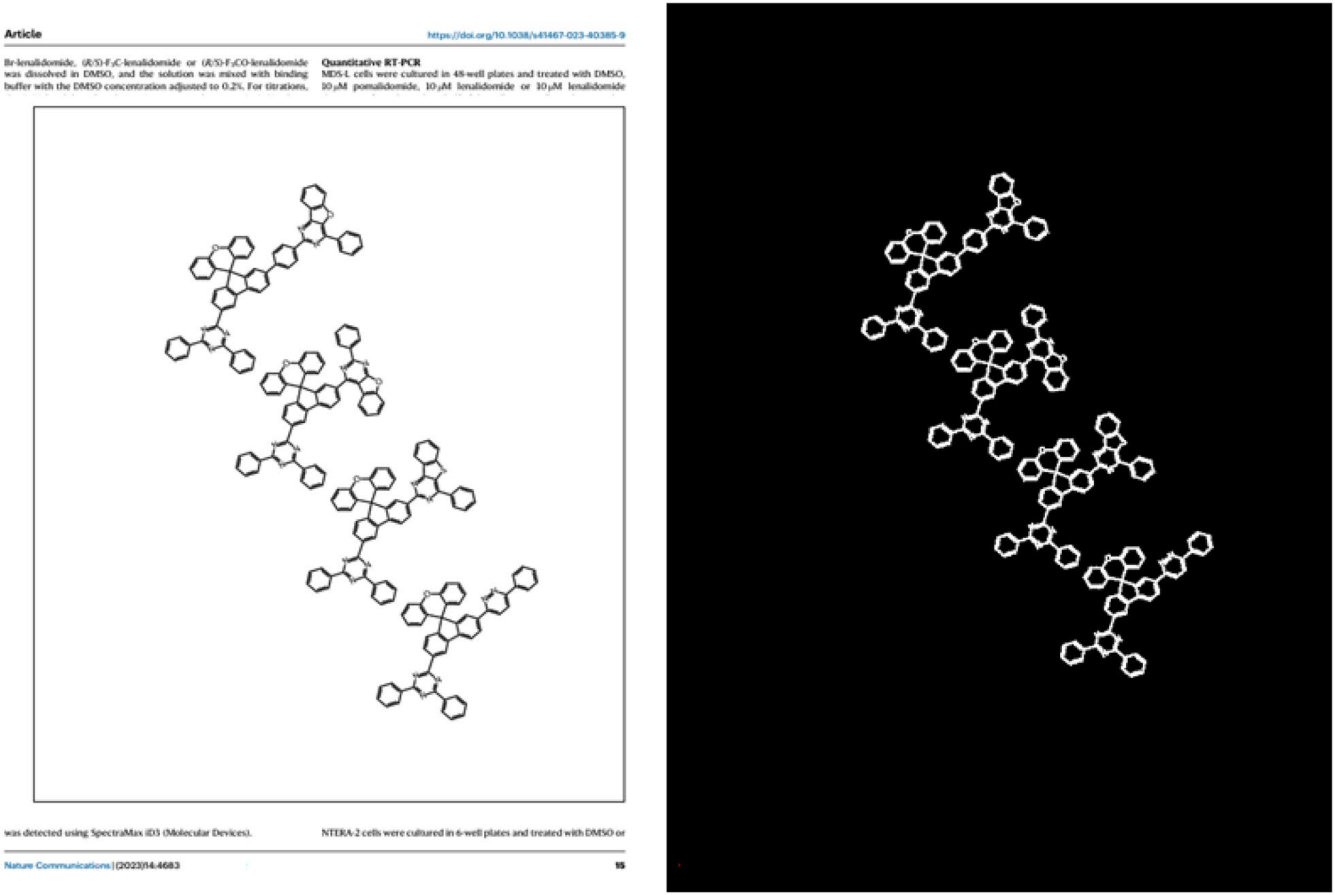
A training example of synthetic page and mask page

Specifically, RDkit [33] (https://www.rdkit.org/) was utilized to convert molecules into images, which were then randomly rotated within a range of 0 to 30 degrees and superimposed onto the page. It was ensured that the molecular structures were completely placed on the page against a white background. To accommodate the varying quality of molecular structure images in real-world scenarios, the thickness of the molecular lines was adjusted during the molecule construction process. Additionally, grid lines were added around the molecular structures to simulate occasional disruptions caused by table grid lines in the molecular structure images. Corresponding mask pages were generated for each covered page, with pixels representing the molecules set to 1 and other areas set to 0. An example from the training data is shown in Fig. 4. The training data comprises approximately 30,784 pairs.

Instead of training from scratch, the above synthetic data was utilized to refine the ChemSAM model derived from SAM-B, previously trained on the SA-1B dataset, which contains over 1 billion masks and 11 million images [22]. In detail, a total of 3784 samples and masks were created for the model’s training.

ChemSAM inherited the predefined hyperparameters from SAM, as established by the Meta AI team. Furthermore, four images per batch were set, taking into account the limited GPU memory resources. A learning rate of 0.001, a learning momentum of 0.9, and an image size of 512 × 512 were established. Details of the configuration settings are available in the supporting materials. The model underwent training on a workstation equipped with an Nvidia 3080 GPU card, 64 GB of RAM, and two Intel(R) Xeon(R) Silver 4114 CPUs. The entire training process took approximately 28 days in total.

### Post-processing

Three common problems exist within our ChemSAM molecular structure segmentation. The first problem is that chemical structures are often displayed within tables, particularly in patents and scientific journals. Occasionally, the table lines overlap with chemical structures. ChemSAM may consider the table lines as part of the molecular structures. A line removal workflow was introduced to eliminate long, straight horizontal and vertical lines in both images and predicted masks, utilizing the Hough transform [34].

The second problem involves predicted masks not perfectly covering the original chemical structures, sometimes resulting in incomplete coverage or the addition of extraneous masks, as indicated in Fig. 1B. To ensure molecular integrity, a custom mask updating algorithm was developed. This algorithm overlays the predicted masks on the scaled images, adjusting them to add necessary coverage for all chemical structures and remove extraneous non-chemical masks. The workflow for updating predicted masks begins with binarizing the input image using an adaptive threshold, which is determined by the mean value of the image. Binarization ensures that any non-white backgrounds or artifacts from low-quality scans are filtered out. Subsequently, dilation is applied to the binary image of chemical structures to connect and close gaps, such as those between atomic element symbols and adjacent bonds, through the expansion of non-white pixels. The scanning matrix used for dilation is a scaled square, its size dependent on the dimensions of the input image. The pixel-wise masks obtained from ChemSAM are binarized to eliminate low-confidence pixels, followed by the aforementioned post-processing steps, including table line removal and binary dilation.

**Fig. 5:**
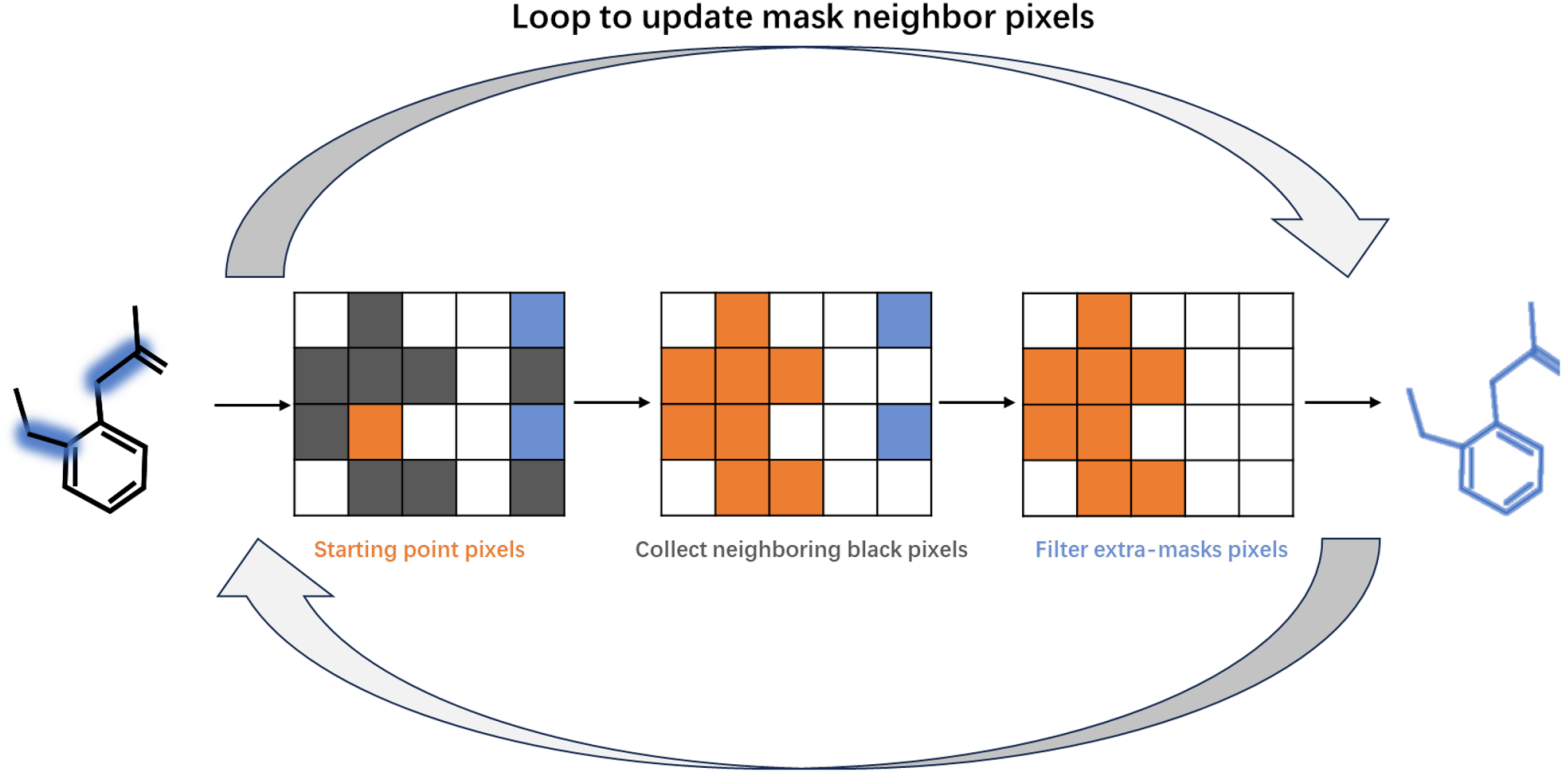
The mask update process. The update process consists of two steps: masks addition and deletion. Search to add black pixels from neighbor neighbors and delete mask pixels that are further away

**Fig. 6:**
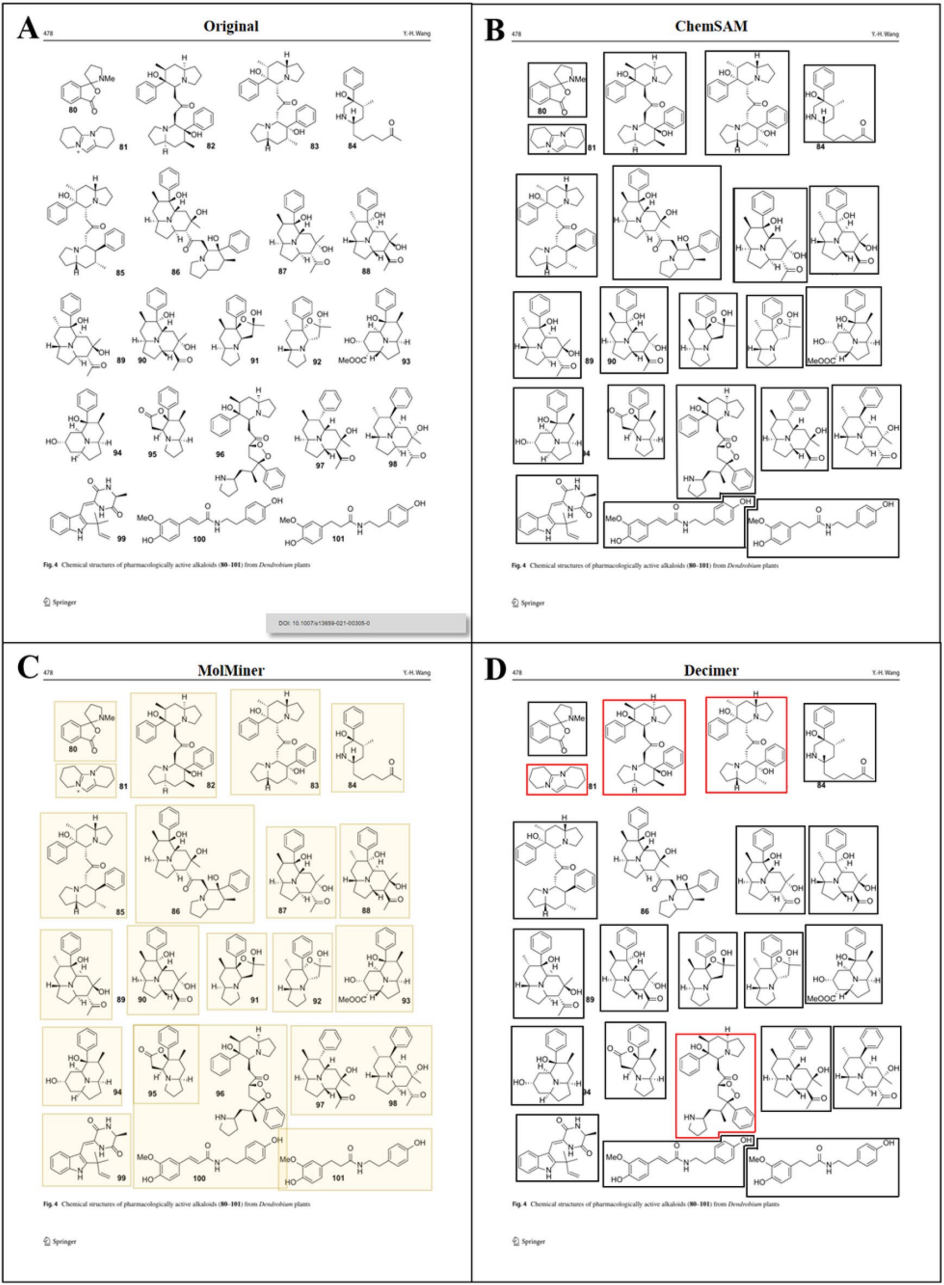
Case of one scanned journal page from Wang [35]. **A** is the original page, **B** is output of ChemSAM model; **C** is output of MolMiner model; **D** is output of DECIMER model

Then, the dilated masks acted as initial points for the subsequent updating process, involving the addition and deletion of masks. By overlaying the mask and pixel pages, pixels corresponding to these initial points are iteratively examined, as displayed in Fig. 5. Starting from each pixel, the process performs a neighbor search, collecting adjacent black pixels. The search persists until a non-black pixel is encountered or no additional adjacent pixels are discovered. Masks retain the same positions as the added adjacent pixels and their corresponding initial points. This mask-adding step enables the capture of all pixels within the intact molecular structure, provided that a single mask prediction is successful. Note that the mask’s background is black, and white represents the predicted structural mask, whereas this is reversed for most patents and papers, which feature a white background and black text. Following the pixel-level addition process, the molecular pixel masks are reduced in size, with efforts made to maintain the same number of molecular pixels by aligning the input with the mask page, as shown in Fig. 1C. Additionally, extra masks are filtered out by removing areas too small to be considered structures, counting the number of pixels in a contiguous area and deeming it a non-structure if the pixel count falls below a threshold (400 pixels at 512 × 512 height-width). Individual entities, defined as single, contiguous groups of positively predicted pixels within the refined masks, are presumed to contain single structures. These are utilized to crop structures from the original inputs, yielding a collection of individual structure images, as indicated in Fig. 6B.

Last but not least is the challenge of false positive detection. ChemSAM can automatically avoid many common cases, such as Western blot figures and statistical analysis charts. However, in some special cases, as displayed in Additional file 1: Fig. S2, ChemSAM still fails to separate the non-chemical parts. Indeed, the chemical parts are tightly connected to the non-chemical parts, or the chemical structures overlay the non-chemical parts. The failures can be attributed to the absence of similar data in our synthetic training dataset. Currently, preparing this training data presents challenges. We developed an in-house filter model, also a ViT-based encoder-decoder, that takes the segmented results as input to determine whether an image represents a chemical structure. If the input does not represent a molecular structure, the image is skipped and labeled as non-molecule. Otherwise, the image is converted into an RDkit 2D molecular graph [33] and saved as an SDF file or in other specified formats.

## Result and discussion

In evaluating the segmentation capabilities of MolMiner-ImgDet [20], DECIMER [18], and ChemSAM, we reference several considerations due to the varied availability of model implementations and the specific challenges presented by chemical structure segmentation. These considerations include the completeness of segmentation, the proportion of structures accurately segmented from the document layout, the recognition rate of colored structures, and the proportion of non-structural elements. We aimed to compare the segmentation capabilities of and ChemSAM. However, given MolMiner-ImgDet’s model has not been officially released, our evaluation primarily focuses on segmentation completeness for this model, using cases from its published article. For DECIMER and ChemSAM, we extend our assessment to include additional aspects such as the rate of correctly identified structures, the proportion of non-structural elements, and the accuracy in recognizing colored structures where applicable. These references serve not as strict standards but as guidelines to gauge the performance of each model in handling the intricate task of chemical structure segmentation.

As illustrated in Fig. 5, MolMiner exhibits inadequacies in accurately segmenting individual molecules, leading to instances where two or more molecules are erroneously grouped within a single image fragment. These inaccuracies can potentially introduce undesired complications in subsequent recognition steps, necessitating additional efforts to re-segment images containing multiple molecules into individual patterns. This challenge was aptly characterized in the original text as MolMiner’s weakness in handling crowded layout segmentation.

In contrast, the DECIMER model’s segmentation capability is relatively robust. However, it failed to accurately recognize and segment the 86 compounds depicted in the image. Unexpectedly, four instances of segmentation errors were noted (highlighted in red boxes), with chiral hydrogens misidentified as methyl groups and charge symbols being overlooked. Notably, DECIMER’s output omitted all compound index labels. Thus, the model overlooked the chiral hydrogens and charge symbols for compounds 82, 83, and 96, possibly due to their structural spacing in the image.

In this instance, ChemSAM demonstrated flawless segmentation. During the recognition process, the ChemSAM model meticulously explores the pixels surrounding the structures. Consequently, labels for compounds 80 and 90, located in close proximity to their respective structures, were accurately retained. Furthermore, ChemSAM accurately identified chiral hydrogens and positive charge symbols.

To systematically evaluate the ability to segment from patents and papers, we constructed a chemical structure dataset as a testing benchmark, gathering 25 recent journal articles and patents from the past 2 years. DECIMER and ChemSAM were evaluated using this benchmark, with the results presented in Fig. 6. Detailed performance metrics are available in Additional file 1: Table S1. Regarding completeness, ChemSAM achieved an astounding accuracy rate of 98.43%, whereas DECIMER’s performance, aligning with its published results, reached a rate of 90.15% on the testing benchmark. However, it is notable that DECIMER exhibited a 2.54% redundancy in its recognition process, which could lead to misalignment between identified structures and their annotated positions, presenting challenges in biochemical data entry.

**Fig. 7:**
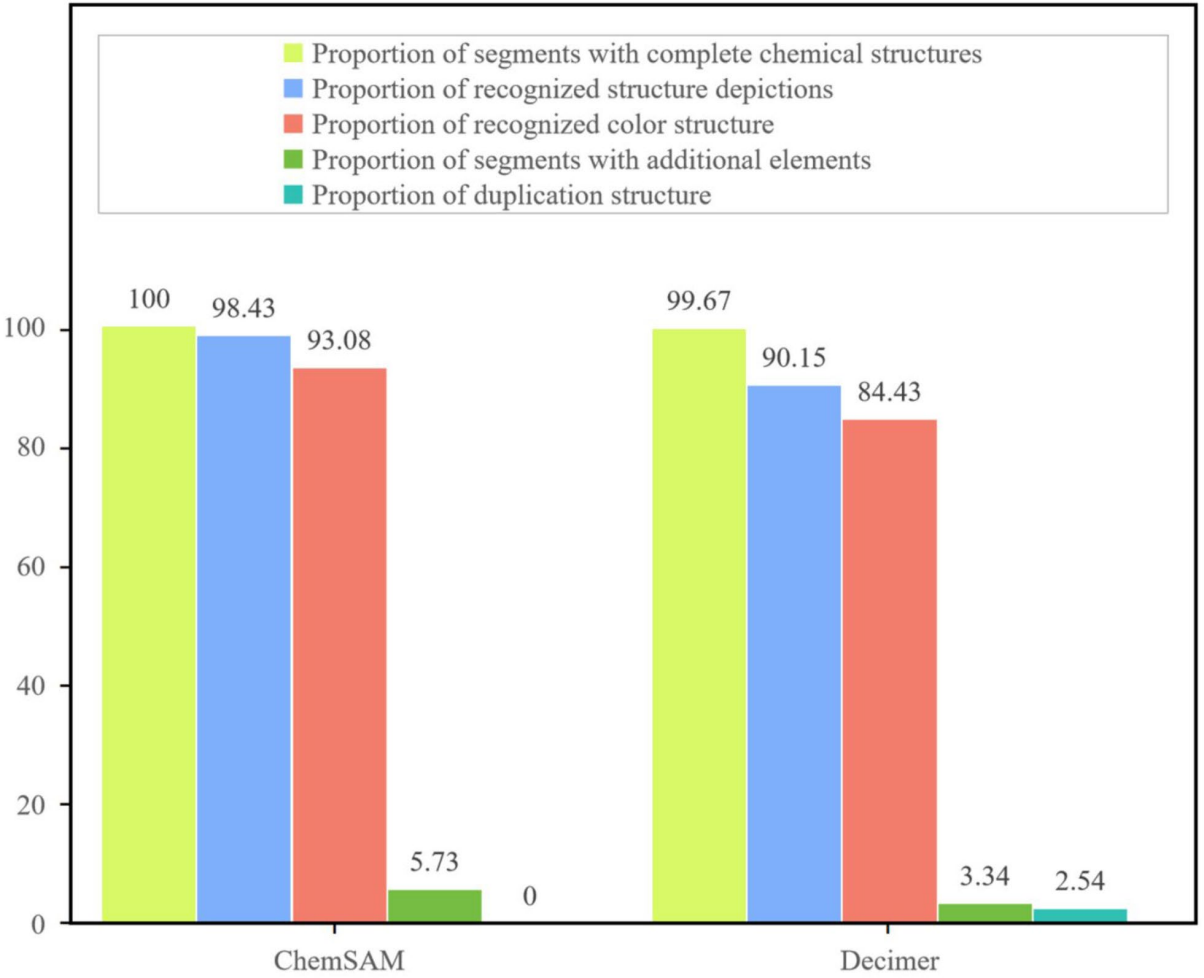
The histogram statistical results of the benchmark data set

**Fig. 8:**
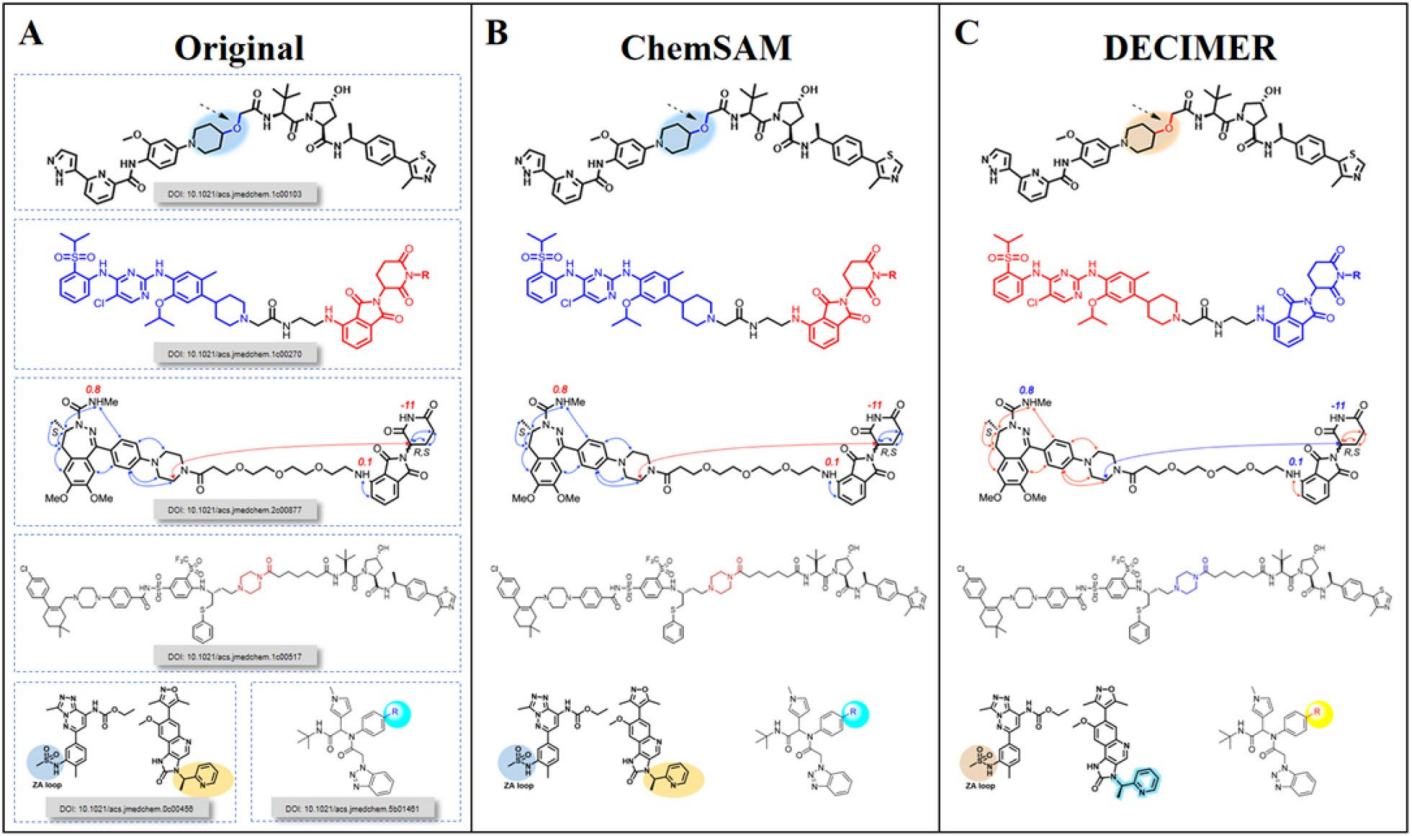
Colorful molecular structure images detection and segments

Additionally, in recognizing color structures or chemical structures against colored backgrounds, ChemSAM’s success rate exceeds DECIMER’s by nearly 10 percentage points, as shown in Fig. 7. Specifically, ChemSAM can directly recognize color images and uses an adjusted mask to crop the original image, ensuring consistency between the output and the source. In contrast, DECIMER inverts image colors during the recognition process, leading to noticeable changes in some color nuances, as illustrated in Fig. 8.

Furthermore, ChemSAM exhibits a notable advantage in processing grayscale patent documents, particularly in recognizing Markush structures within patents, as detailed in the supporting information. This advantage may stem from the unique nature of Markush structures, which differ from complete chemical structures, leading to a decrease in DECIMER’s recognition performance. It is important to note that although Markush structures are not complete chemical structures, they are commonly employed in patents and journal articles to denote a range of compounds. Therefore, the capability to segment and identify Markush structures is essential for extracting chemical information from documents, and addressing Markush structures poses a significant challenge in “figure to mol” translation.

**Fig. 9:**
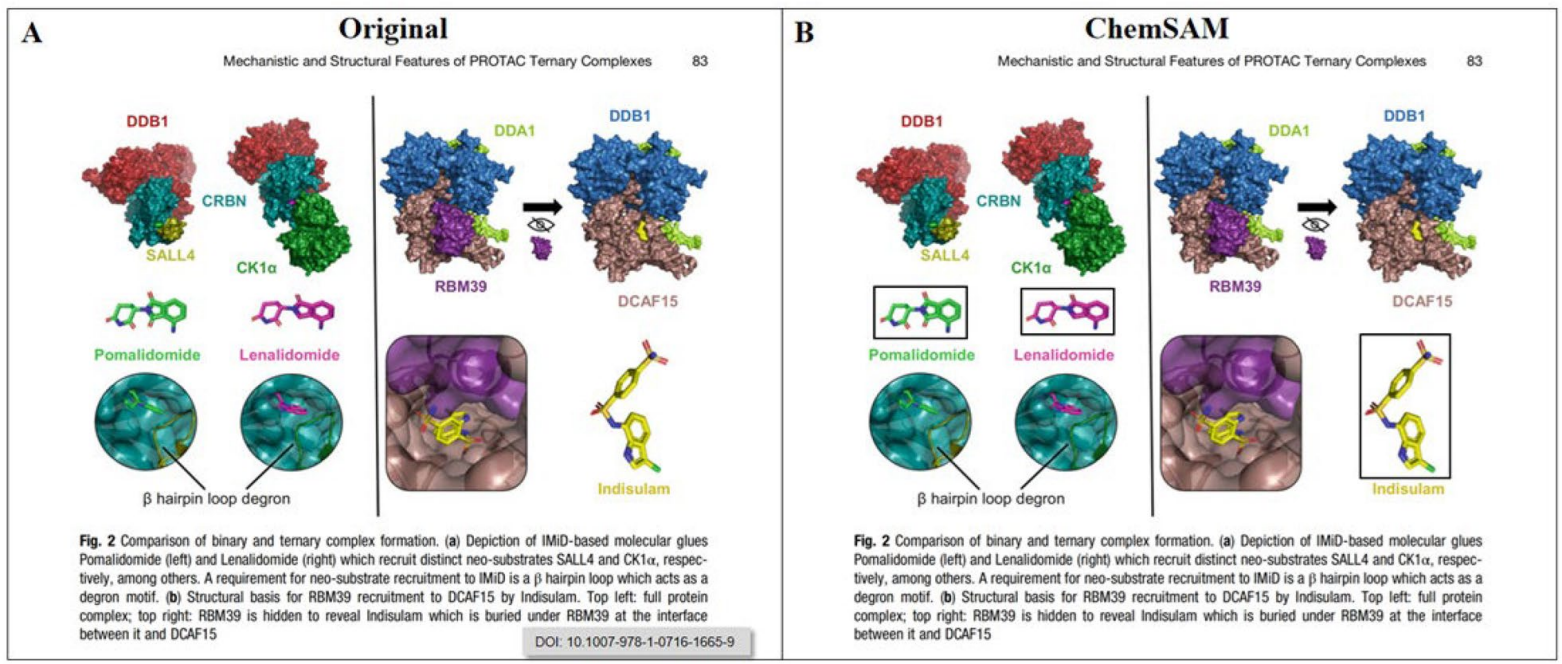
3D molecular structure image detection and segment

Unlike DECIMER segmentation, which tends to include non-molecular structure parts in its generated mask areas due to its reliance on so-called region proposal networks for generating regions of interest, ChemSAM predicts the chemical structure mask directly at the pixel level, aiming to identify an equal quantity of pixels that belong to chemical structures. Furthermore, ChemSAM can segment 3D molecular images, as displayed in Fig. 9, despite the absence of 3D molecular structures in its training data.

ChemSAM outperforms the open-source solution, DECIMER. It can detect and separate many molecules that DECIMER cannot, with examples displayed in Additional file 1: Fig. S3. When the volume of segmentation work is manageable, missing molecular structures may be segmented by hand. Otherwise, completing the task quickly becomes impractical, given the need to segment hundreds of patents or papers and convert them into chemical datasets. This is especially true for patents containing up to one thousand pages. ChemSAM accepts various file formats as inputs, including PNG, JPG, SVG, and PDF.

In summary, our proposed method achieves state-of-the-art results, demonstrating its effectiveness in accurately segmenting chemical structures from text-based sources. Particularly in terms of completeness and accuracy, it significantly outperforms DECIMER. Although it slightly lags behind DECIMER in segmenting chemical structures with additional elements, this gap is negligible compared to other performance indicators. However, these additional elements may not pose a problem in the subsequent process of translating segmented figures into molecules. For instance, the published tool Molscribe [16] can readily translate chemical figures containing additional elements into target molecules (Fig. 9).

## Conclusion

Chemical structure segmentation is a crucial step in chemical structure recognition. Most research works have focused on the step of translating molecular figures into molecular structures, assuming the inputs are suitably prepared containing a single chemical structure. However, in practice, developing an automatic method to segment each molecular structure as an independent image is challenging due to varied drawing styles and dense arrangements. We propose a deep learning approach to chemical structure segmentation, utilizing a vision transformer model to discern the structural patterns of chemical compounds from their graphical representations. Our method achieves state-of-the-art results on publicly available benchmark datasets, demonstrating its effectiveness in accurately segmenting chemical structures from text-based sources. Furthermore, our findings indicate that this method is highly effective in extracting chemical structures from text-based sources, with potential applications across various domains in cheminformatics. Overall, this study contributes to advancing more accurate and efficient chemical structure segmentation methods, with significant implications for drug discovery, chemical synthesis, and broader chemical research areas.

### Supplementary Information

**Additional file 1: Figure S1.** The distribution of complete atomic distribution of data. **Figure S2.** Example of failed to split from non-chemical part. **Figure S3.** Example of color structure recognition. **Table S1.** Benchmark data test on ChemSAM and DECIMER.

## Data Availability

The implementation details of the model training and the calculation of evaluation metrics are openly accessible for review and replication. Interested parties can find the source code and relevant documentation in the project’s GitHub repository [21]. Meanwhile, the training data crucial to this study’s findings are publicly available for academic scrutiny and further research. The dataset encompasses a wide array of molecular structures and is instrumental in the development and refinement of the ChemSAM model. Researchers and practitioners can access and download the dataset through the Google Drive [36]. Additionally, the ChemSAM model has been successfully applied in the PROTACT database project [37], demonstrating its practical utility in real-world cheminformatics applications. The project showcases the model’s capability to accurately segment and identify chemical structures within a complex database environment, underscoring ChemSAM’s potential for broad adoption in chemical research and drug discovery endeavors.

## References

[1] Park J, Rosania GR, Shedden KA, Nguyen M, Lyu N, Saitou K (2009). Automated extraction of chemical structure information from digital raster images. Chem Cent J.

[2] Ibison P, Jacquot M, Kam F, Neville A, Simpson RW, Tonnelier C, Venczel T, Johnson AP (1993). Chemical literature data extraction: the CLiDE project. J Chem Inf Comput Sci.

[3] Sadawi NM, Sexton AP, Sorge V (2012) Chemical structure recognition: a rule-based approach. In: Document recognition and retrieval XIX. SPIE, pp 101–109

[4] Valko AT, Johnson AP (2009). CLiDE Pro: the latest generation of CLiDE, a tool for optical chemical structure recognition. J Chem Inf Model.

[5] McDaniel JR, Balmuth JR (1992). Kekule: OCR-optical chemical (structure) recognition. J Chem Inf Comput Sci.

[6] Frasconi P, Gabbrielli F, Lippi M, Marinai S (2014). Markov logic networks for optical chemical structure recognition. J Chem Inf Model.

[7] Casey R, Boyer S, Healey P, Miller A, Oudot B, Zilles K (1993) Optical recognition of chemical graphics. In: Proceedings of 2nd international conference on document analysis and recognition (ICDAR'93). IEEE, pp 627–631

[8] Filippov IV, Nicklaus MC (2009). Optical structure recognition software to recover chemical information: OSRA, an open source solution. J Chem Inf Model.

[9] Algorri M-E, Zimmermann M, Friedrich CM, Akle S, Hofmann-Apitius M (2007) Reconstruction of chemical molecules from images. In: 2007 29th annual international conference of the IEEE engineering in medicine and biology society. IEEE, pp 4609–4612 10.1109/IEMBS.2007.4353366 18003032

[10] Rajan K, Brinkhaus HO, Agea MI, Zielesny A, Steinbeck C (2023) DECIMER. ai-An open platform for automated optical chemical structure identification, segmentation and recognition in scientific publications. Nat Commun 14(5045):1-18. 10.1038/s41467-023-40782-0 10.1038/s41467-023-40782-0 PMC10439916 37598180

[11] Clevert D-A, Le T, Winter R, Montanari F (2021). Img2Mol—accurate SMILES recognition from molecular graphical depictions. Chem Sci.

[12] Xu Z, Li J, Yang Z, Li S, Li H (2022). SwinOCSR: end-to-end optical chemical structure recognition using a Swin transformer. J Cheminform.

[13] Beard EJ, Cole JM (2020). ChemSchematicResolver: a toolkit to decode 2D chemical diagrams with labels and R-groups into annotated chemical named entities. J Chem Inf Model.

[14] Yoo S, Kwon O, Lee H (2022) Image-to-graph transformers for chemical structure recognition. In: ICASSP 2022–2022 IEEE international conference on acoustics, speech and signal processing (ICASSP). IEEE, pp 3393–3397

[15] Staker J, Marshall K, Abel R, McQuaw CM (2019). Molecular structure extraction from documents using deep learning. J Chem Inf Model.

[16] Qian Y, Guo J, Tu Z, Li Z, Coley CW, Barzilay RJ (2023). MolScribe: robust molecular structure recognition with image-to-graph generation. J Chem Inf Model.

[17] Ronneberger O, Fischer P, Brox T (2015) U-net: convolutional networks for biomedical image segmentation. In: Medical image computing and computer-assisted intervention—MICCAI 2015: 18th international conference, Munich, Germany, October 5–9, 2015, proceedings, part III 18. Springer, pp 234–241

[18] Rajan K, Brinkhaus HO, Sorokina M, Zielesny A, Steinbeck C (2021). DECIMER-segmentation: automated extraction of chemical structure depictions from scientific literature. J Cheminform.

[19] He K, Gkioxari G, Dollár P, Girshick R (2017) Mask r-cnn. In: Proceedings of the IEEE international conference on computer vision, pp 2961–2969

[20] Xu Y, Xiao J, Chou C-H, Zhang J, Zhu J, Hu Q, Li H, Han N, Liu B, Zhang S (2022). MolMiner: you only look once for chemical structure recognition. J Chem Inf Model.

[21] ChemSAM project. https://github.com/mindrank-ai/ChemSAM/tree/master

[22] Kirillov A, Mintun E, Ravi N, Mao H, Rolland C, Gustafson L, Xiao T, Whitehead S, Berg AC, Lo W-Y (2023) Segment anything. arXiv:2304.02643

[23] Paszke A, Gross S, Massa F, Lerer A, Bradbury J, Chanan G, Killeen T, Lin Z, Gimelshein N, Antiga L (2019) Pytorch: an imperative style, high-performance deep learning library. In: Advances in neural information processing systems, 32

[24] Dosovitskiy A, Beyer L, Kolesnikov A, Weissenborn D, Zhai X, Unterthiner T, Dehghani M, Minderer M, Heigold G, Gelly S (2020) An image is worth 16x16 words: transformers for image recognition at scale. arXiv:2010.11929

[25] He K, Chen X, Xie S, Li Y, Dollár P, Girshick R (2022) Masked autoencoders are scalable vision learners. In: Proceedings of the IEEE/CVF conference on computer vision and pattern recognition, pp 16000–16009

[26] Tancik M, Srinivasan P, Mildenhall B, Fridovich-Keil S, Raghavan N, Singhal U, Ramamoorthi R, Barron J, Ng R (2020). Fourier features let networks learn high frequency functions in low dimensional domains. Adv Neural Inf Process Syst.

[27] Radford A, Kim JW, Hallacy C, Ramesh A, Goh G, Agarwal S, Sastry G, Askell A, Mishkin P, Clark J (2021) Learning transferable visual models from natural language supervision. In: International conference on machine learning. PMLR, pp 8748–8763

[28] Carion N, Massa F, Synnaeve G, Usunier N, Kirillov A, Zagoruyko S (2020) End-to-end object detection with transformers. In: European conference on computer vision. Springer, pp 213–229

[29] Vaswani A, Shazeer N, Parmar N, Uszkoreit J, Jones L, Gomez AN, Kaiser Ł, Polosukhin I (2017). Attention is all you need. Advances in neural information processing systems.

[30] Nair V, Hinton GE (2010) Rectified linear units improve restricted Boltzmann machines. In: Proceedings of the 27th international conference on machine learning (ICML-10), pp 807–814

[31] Marco AC, Myers A, Graham SJ, D'Agostino P, Apple K (2015) The USPTO patent assignment dataset: descriptions and analysis

[32] Sterling T, Irwin JJ (2015). ZINC 15–ligand discovery for everyone. J Chem Inf Model.

[33] Landrum G, et al. RDKit: open-source cheminformatics software. 2016. https://www.rdkit.org/, https://github.com/rdkit/rdkit. Accessed 16 October 2023

[34] Galamhos C, Matas J, Kittler J (1999) Progressive probabilistic Hough transform for line detection. In: Proceedings 1999 IEEE computer society conference on computer vision and pattern recognition (Cat No PR00149). IEEE, pp 554–560

[35] Wang Y-H (2021). Bioprospecting: traditional uses and pharmacologically active constituents of Dendrobium plants for dermatological disorders: a review. Nat Prod Bioprospect.

[36] Dataset. https://drive.google.com/file/d/1RZBpDk4EkM7UI9QDV5gdP2x2iVmqtlR5/view?usp=drive_link

[37] PROTACT database project. http://newblock.xq200.com

