# Supplementary material for: Automated molecular structure segmentation from documents using ChemSAM
Source: J Cheminform. 2024 Mar 12;16:29. doi: 10.1186/s13321-024-00823-2 (PMC10935819; doi:10.1186/s13321-024-00823-2)
Supplement: Supplementary file 1 — Additional file 1: Figure S1. The distribution of complete atomic distribution of data. Figure S2. Example of failed to split from non-chemical part. Figure S3. Example of color structure recognition. Table S1. Benchmark data test on ChemSAM and DECIMER. [file 13321_2024_823_MOESM1_ESM.docx]

**Additional file 1**

**Fig.S1** The distribution of complete atomic distribution of data.


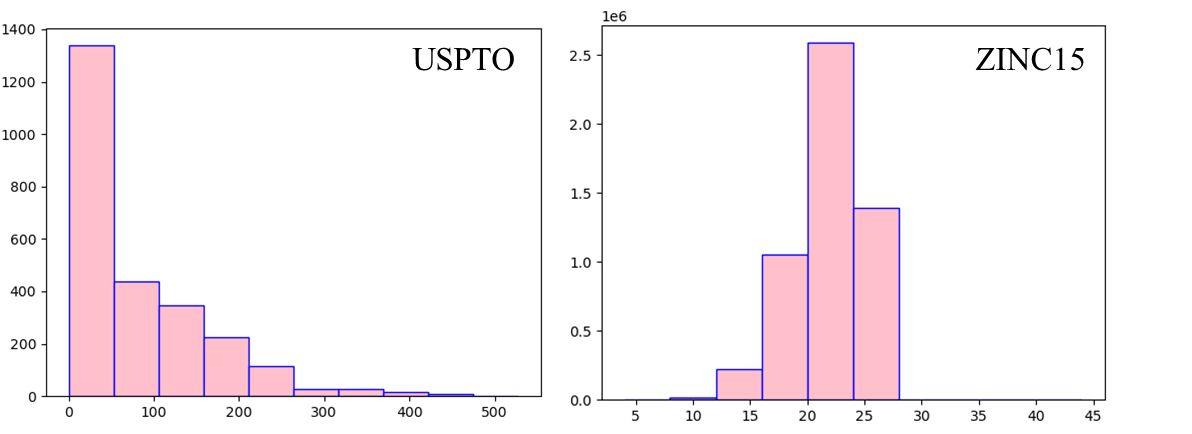


Among them, the abscissa represents the number of atoms in the molecule, and the ordinate represents the number of molecules.

Fig. S2 Example of failed to split from non-chemical part


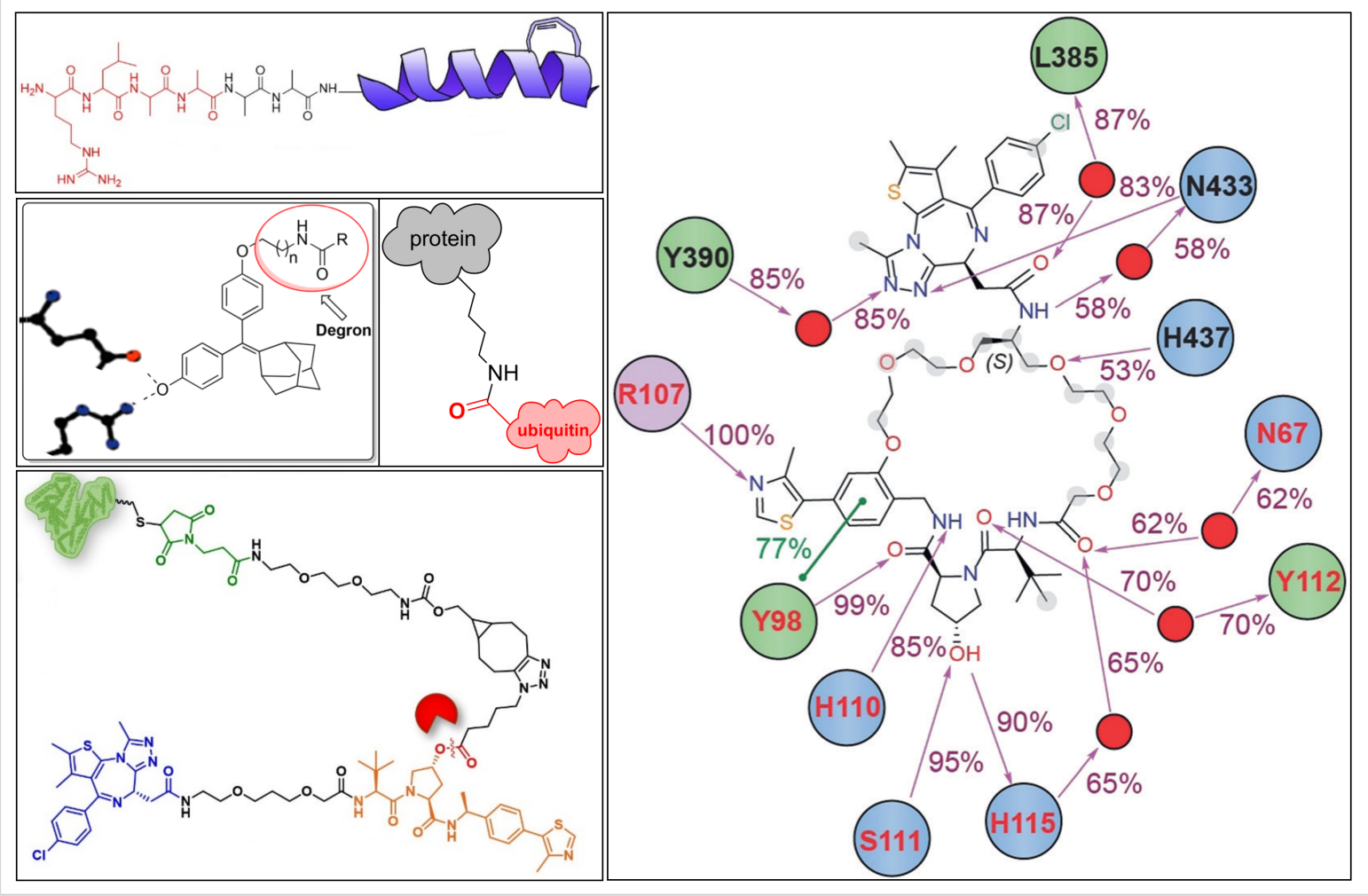


Fig. S3 Example of color structure recognition


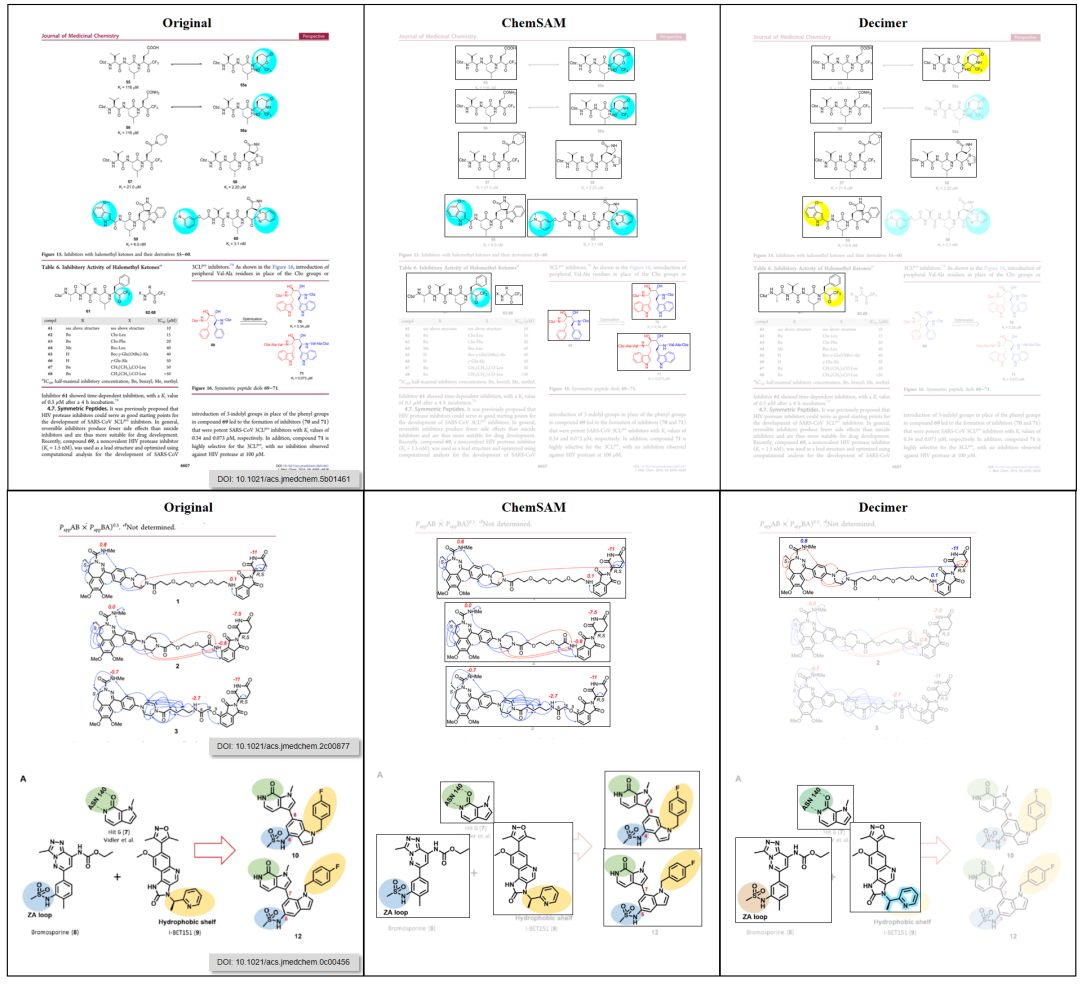


**Table S1. Benchmark data test on ChemSAM and DECIMER**

|  | Original | | ChemSAM | | | | | DECIMER | | | | |
| --- | --- | --- | --- | --- | --- | --- | --- | --- | --- | --- | --- | --- |
| DOI | TS*^a^* | TC*^b^* | TS*^a^* | TC*^b^* | SCN*^c^* | NS*^d^* | ND*^e^* | TS*^a^* | TC*^b^* | SCN*^c^* | NS*^d^* | ND*^e^* |
| acs.jmedchem.0c00456 | 76 | 10 | 73 | 8 | 5 | 2 | 0 | 71 | 6 | 3 | 2 | 0 |
| acs.jmedchem.0c00830 | 81 | 18 | 80 | 17 | 0 | 0 | 0 | 74 | 11 | 0 | 0 | 2 |
| acs.jmedchem.0c00919 | 100 | 81 | 94 | 75 | 14 | 0 | 0 | 97 | 77 | 8 | 1 | 0 |
| acs.jmedchem.0c01127 | 44 | 1 | 44 | 1 | 5 | 0 | 0 | 43 | 0 | 2 | 0 | 0 |
| acs.jmedchem.0c01947 | 3 | 0 | 3 | 0 | 0 | 0 | 0 | 3 | 0 | 3 | 0 | 1 |
| acs.jmedchem.1c00103 | 23 | 1 | 22 | 1 | 1 | 0 | 0 | 23 | 1 | 1 | 0 | 1 |
| acs.jmedchem.1c00270 | 65 | 22 | 61 | 18 | 8 | 1 | 0 | 64 | 21 | 8 | 0 | 0 |
| acs.jmedchem.1c00517 | 28 | 3 | 27 | 2 | 3 | 0 | 0 | 27 | 2 | 3 | 0 | 2 |
| acs.jmedchem.1c00629 | 133 | 41 | 132 | 41 | 10 | 1 | 0 | 125 | 35 | 7 | 0 | 0 |
| acs.jmedchem.1c00958 | 22 | 1 | 22 | 1 | 2 | 0 | 0 | 21 | 1 | 1 | 1 | 0 |
| acs.jmedchem.2c00877 | 7 | 3 | 6 | 3 | 0 | 0 | 0 | 5 | 1 | 0 | 0 | 0 |
| acs.jmedchem.2c01300 | 56 | 6 | 56 | 6 | 7 | 0 | 0 | 53 | 4 | 4 | 1 | 0 |
| acs.jmedchem.3c00823 | 8 | 2 | 8 | 2 | 0 | 0 | 0 | 7 | 1 | 0 | 1 | 0 |
| acs.jmedchem.5b00127 | 50 | 1 | 48 | 1 | 0 | 0 | 0 | 44 | 1 | 0 | 0 | 0 |
| acs.jmedchem.5b01461 | 176 | 92 | 171 | 86 | 8 | 2 | 0 | 157 | 76 | 4 | 1 | 1 |
| acs.jmedchem.9b01180 | 10 | 7 | 10 | 7 | 0 | 0 | 0 | 10 | 7 | 0 | 1 | 0 |
| ange.201501394 | 15 | 0 | 15 | 0 | 1 | 1 | 0 | 15 | 0 | 0 | 0 | 0 |
| anie.201206231 | 4 | 0 | 4 | 0 | 0 | 0 | 0 | 4 | 0 | 0 | 0 | 0 |
| anie.201601091 | 40 | 0 | 39 | 0 | 1 | 0 | 0 | 40 | 0 | 1 | 0 | 0 |
| WO2023077441A1 | 129 | 0 | 128 | 0 | 3 | 0 | 0 | 118 | 0 | 3 | 0 | 5 |
| WO2022098544A1 | 497 | 0 | 488 | 0 | 35 | 6 | 0 | 356 | 0 | 12 | 0 | 16 |
| WO2021146536A1 | 383 | 0 | 382 | 0 | 1 | 0 | 0 | 369 | 0 | 1 | 0 | 20 |
| US20210147383A1 | 294 | 0 | 293 | 0 | 0 | 2 | 0 | 279 | 0 | 0 | 0 | 0 |
| CN116354927A | 102 | 0 | 102 | 0 | 11 | 0 | 0 | 97 | 0 | 10 | 0 | 0 |
| CN115724836A | 273 | 0 | 270 | 0 | 35 | 0 | 0 | 259 | 0 | 9 | 0 | 10 |

*^a^*The total number of structures(TS) after artificially removing fragments with less than two rings. *^b^*The total number of color structure (TC) which contain color element or color background. *^c^*The total number of structure which contain non-structure (SCN) element. *^d^*The total number of non-structure output. *^e^*The total number of duplicate output.
